# Guidelines and Best Practices for the Use of Targeted Maximum Likelihood and Machine Learning When Estimating Causal Effects of Exposures on Time‐To‐Event Outcomes

**DOI:** 10.1002/sim.70034

**Published:** 2025-03-13

**Authors:** Denis Talbot, Awa Diop, Miceline Mésidor, Yohann Chiu, Caroline Sirois, Andrew J. Spieker, Antoine Pariente, Pernelle Noize, Marc Simard, Miguel Angel Luque Fernandez, Michael Schomaker, Kenji Fujita, Danijela Gnjidic, Mireille E. Schnitzer

**Affiliations:** ^1^ Département de Médecine Sociale et Préventive Université Laval Québec Canada; ^2^ Axe Santé des Populations et Pratiques Optimales en Santé Centre de Recherche du CHU de Québec – Université Laval Québec Canada; ^3^ Faculté de Pharmacie Université Laval Québec Canada; ^4^ Bureau d'information et d'études en santé des populations Institut national de santé publique du Québec Québec Canada; ^5^ VITAM—Centre de Recherche en Santé Durable Centre Intégré de Santé et de Services Sociaux de la Capitale Nationale Québec Canada; ^6^ Department of Biostatistics Vanderbilt University Medical Center Nashville Tennessee USA; ^7^ University of Bordeaux Bordeaux France; ^8^ Inequalities in Cancer Outcomes Network London School of Hygiene and Tropical Medicine London UK; ^9^ Department of Statistics and Operations Research University of Granada Granada Spain; ^10^ Ludwig‐Maximilans‐Universität München Germany; ^11^ Kolling Institute University of Sydney Sydney New South Wales Australia; ^12^ Northern Sydney Local Health District Sydney New South Wales Australia; ^13^ School of Pharmacy and Faculty of Medicine and Health University of Sydney Sydney New South Wales Australia; ^14^ Faculté de Pharmacie Université de Montréal Québec Canada; ^15^ Département de Médecine Sociale et Préventive Université de Montréal Québec Canada

**Keywords:** causal inference, double robustness, machine learning, observational studies, survival analysis, targeted maximum likelihood estimation

## Abstract

Targeted maximum likelihood estimation (TMLE) is an increasingly popular framework for the estimation of causal effects. It requires modeling both the exposure and outcome but is doubly robust in the sense that it is valid if at least one of these models is correctly specified. In addition, TMLE allows for flexible modeling of both the exposure and outcome with machine learning methods. This provides better control for measured confounders since the model specification automatically adapts to the data, instead of needing to be specified by the analyst *a priori*. Despite these methodological advantages, TMLE remains less popular than alternatives in part because of its less accessible theory and implementation. While some tutorials have been proposed, none address the case of a time‐to‐event outcome. This tutorial provides a detailed step‐by‐step explanation of the implementation of TMLE for estimating the effect of a point binary or multilevel exposure on a time‐to‐event outcome, modeled as counterfactual survival curves and causal hazard ratios. The tutorial also provides guidelines on how best to use TMLE in practice, including aspects related to study design, choice of covariates, controlling biases and use of machine learning. R‐code is provided to illustrate each step using simulated data ( https://github.com/detal9/SurvTMLE). To facilitate implementation, a general R function implementing TMLE with options to use machine learning is also provided. The method is illustrated in a real‐data analysis concerning the effectiveness of statins for the prevention of a first cardiovascular disease among older adults in Québec, Canada, between 2013 and 2018.

**Abbreviation**
TMLE: targeted maximum likelihood estimation

## 1 Introduction

Much of pharmacoepidemiological research is concerned with exposure (or treatment) effect estimation using observational data. When there are systematic differences in outcome predictive characteristics between exposure groups, adequate control for confounding bias is paramount. Traditional survival analysis modeling, such as Cox proportional hazards models, can model the effect of a point‐exposure on a time‐to‐event outcome, conditional on confounding variables. More recently, causal inference methods for survival analysis have allowed for counterfactual definitions of causal effects, independent of a true or working outcome model. These causal parameters can be estimated by any number of methods. For example, the popular inverse probability of treatment weighting (IPTW) can both adjust for exposure group confounders through propensity scores while simultaneously allowing for adjustment of longitudinal missing‐at‐random censoring. However, limitations of IPTW, such as suboptimal efficiency and instability, have led to the development of more robust approaches to the estimation of causal inference parameters.

One such approach is targeted maximum likelihood estimation (TMLE) [1], an estimation framework for efficient estimation that can readily incorporate machine learning. TMLE involves first specifying an initial substitution estimator and then updating it such that the resulting estimator is efficient. Typically, the initial estimator is based on an outcome regression model and the updating step uses information from a propensity score model. Because of its theoretical advantages, TMLE is becoming an increasingly popular analytical approach in pharmacoepidemiology [2]. Multiple simulation studies have been published comparing the empirical performance of TMLE with alternatives under various scenarios [3, 4, 5, 6, 7, 8, 9, 10, 11]. Overall, these simulations showcase the potential benefits of using TMLE to protect against model misspecification bias, especially when combined with machine learning algorithms. However, TMLE remains less frequently used in applied research than simpler alternatives. Some tutorials have been produced to provide intuition on the functioning of TMLE and detail its implementation focusing on the case of a single exposure on a continuous or binary outcome [7, 12, 13, 14, 15]. However, none of the existing tutorials address the case of a time‐to‐event outcome, which is ubiquitous in pharmacoepidemiology.

The objective of this article is to provide a detailed step‐by‐step tutorial on how to estimate the effect of a binary or multilevel exposure measured at a single time point on a time‐to‐event outcome using TMLE. As will be seen, there are several particularities to this context of application. We begin in Section 2 by describing the challenges and advantages of traditional (Cox proportional hazards model) and causal (IPTW, g‐computation, and TMLE) methods. Section 3 gives a motivating example and introduces some notation. Section 4 provides a step‐by‐step tutorial on how to implement TMLE using simulated data without the recourse of an off‐the‐shelf software function or package. The objective is to provide the reader with the intuition on how the TMLE algorithm proceeds to produce estimates and confidence intervals. Section 5 provides tips and guidelines on how best to implement TMLE with a time‐to‐event outcome. We notably address topics such as designs and research questions for which TMLE is appropriate, the choice of the covariates to include and the specification of the exposure and outcome models. We then illustrate the method in a real‐data application concerning the effectiveness of statins for the prevention of a first cardiovascular event among older adults in Section 6. This illustration makes use of an R function that we have developed to simplify the implementation of TMLE for survival analysis. We conclude in Section 7 with a discussion summarizing the main strengths and limitations of TMLE and suggestions on further readings concerning more advanced related topics.

## 2 Background

Various methods can be used to estimate the effect of a point exposure while controlling for confounding. When the outcome is a time‐to‐event variable, the most common option is to include measured confounders as covariates in a Cox proportional hazards model. To control for confounding appropriately, this model must be adequately specified. This means that the relationship between the included variables and the outcome's hazard must be correctly modeled. For example, if there are non‐linear associations or interactions between covariates, exposure and time, they must be modeled. Adequately specifying the Cox model can be a challenging task, and exposure‐confounder interaction terms, while perhaps necessary for appropriate control of confounding, may pose difficulties for interpretation. In addition, hazard ratios inherently suffer from selection bias [16], and thus lack a causal interpretation except under very stringent and unrealistic assumptions [17, 18]. Intuitively, when exposure has an effect on the outcome, people who are more susceptible to the event will have the event sooner in the exposure group that is the most at risk. This differential depletion of susceptible people between groups tends to make the hazard ratio vary in time, even when the exposure effect remains constant [16]. One solution to mitigate this issue is to report adjusted survival curves in addition to hazard ratios [16]. Another challenge with the Cox model is the non‐collapsibility of hazard ratios, which means that the exposure hazard ratio varies according to the variables that are included in the Cox model, even if those variables are not confounders [19]. Finally, the Cox model assumes independent censoring, which precludes the existence of any post‐exposure factor that affects both the time‐to‐event and the time‐to‐censoring mechanisms.

The g‐computation formula (or g‐formula), which is a generalization of the common standardization formula, can also be used to adjust for covariates in survival analysis in order to estimate causal hazard ratios or survival curves. Several ways of implementing the g‐formula are available [20, 21]. A common implementation uses iterated conditional expectations and involves specifying a series of models for the outcome probability at different time‐points as a function of previous covariates and exposure. Unlike the covariate‐adjusted Cox model, g‐computation can adjust for missing‐at‐random (MAR) censoring. This permits for censoring to depend on post‐exposure covariates. However, g‐computation requires that all involved models are correctly specified.

Another alternative to the covariate‐adjusted Cox model are propensity score methods, such as propensity score matching or IPTW [22, 23, 24]. The exposure hazard ratio or survival curves can be estimated in the matched/weighted data, without further adjustment for covariates under the independent censoring assumption [24]. These methods can further be combined with inverse probability of censoring weighting to avoid the independent censoring assumption, instead requiring MAR censoring [25]. Propensity score methods require modeling the exposure according to measured confounders, which is often accomplished using a logistic regression model. In order to control appropriately for confounding, these methods require the correct specification of this exposure model. Unlike the covariate‐adjusted Cox model approach, using a complex model specification does not hinder interpretation when using propensity score methods. As such, using machine learning methods to flexibly specify the exposure model in a data‐adaptive manner may seem like an attractive option [26, 27, 28, 29]. However, propensity score methods can yield biased estimates with high variance when near‐positivity violation problems occur [30]. Informally, near‐positivity violations mean that the probability of receiving or not receiving treatment is close to one for some individuals given their covariates. Using machine learning methods for modeling the exposure has been found to aggravate these positivity problems in some simulation studies [10, 31]. There are also other challenges related to using machine learning methods for modeling the exposure—or for modeling the outcome—such as the suboptimal convergence rate of estimators (i.e., there are estimators whose variance decreases faster with increasing sample size) and the inability to construct valid confidence intervals [32]. In contrast, the Cox model and g‐computation can be less sensitive to near‐positivity violations than inverse probability weighting because of their ability to extrapolate over sparse regions of the data [33].

Finally, one may consider methods that combine both outcome modeling and exposure modeling, such as augmented IPTW [34], doubly‐robust standardization [21], or TMLE [35]. One advantage of these methods is that they are doubly robust approaches; that is, they produce valid estimates if at least one of either the exposure or outcome model is correctly specified. As such, they give two chances to correctly specify a model and obtain valid results, instead of one as in traditional approaches. In addition, these doubly robust methods (combined with a cross‐validation or cross‐fitting procedure) formally allow for flexible modeling of both the exposure and outcome with machine learning methods, limiting the risk of model misspecification and thus residual confounding [32, 36, 37]. In TMLE, this is achieved without limiting ease of interpretation or theoretical properties of the estimator. Some simulation studies have also found that TMLE performs well under near‐positivity violations, even when used together with machine learning [3, 5, 10], although inference under near‐positivity violations is challenging [38, 39]. It is also possible to control for post‐exposure factors that affect both the time‐to‐event and censoring mechanisms, thus alleviating the independent censoring assumption. TMLE can be used to quantify the causal effect of an exposure on a time‐to‐event outcome in various ways, including adjusted survival curves and marginal hazard ratios [35, 40].

Table 1 summarizes the comparison between the various types of approaches for estimating the effect of an exposure on a time‐to‐event outcome.

**TABLE 1:** Summary of approaches for estimating the effect of an exposure on a time‐to‐event outcome.

	Outcome	Exposure	Censoring	Censoring	Requires correct	Machine learning
Method	modeling	modeling	modeling	assumption	specification of	
Cox model	✓			Independent	All models	Not applicable
G‐computation using iterated	✓			MAR	All models	Affects inference
conditional expectations						
Inverse probability of treatment		✓	✓	MAR	All models	Affects inference
and censoring weighting						
Targeted maximum likelihood	✓	✓	✓	MAR	Exposure + censoring or	Can be used without
					outcome	affecting inference or
						interpretation

## 3 Motivational Example and Notation

As a motivational example, we consider the estimation of the effect of statin treatment persistence for at least three months after initiation on a first cardiovascular event among adults aged 66 years or older. To facilitate replication, we consider simulated data for the tutorial presented in Section 4 and only consider real data in the application in Section 6. We will denote by A the exposure, where A=1 represents statin persistence and A=0 represents non‐persistence. While we consider the case of a binary exposure in this example, multilevel categorical exposures are readily accommodated by the procedure we present. The outcome of interest is the time between statin initiation and the occurrence of a first cardiovascular event or death from any cause, T. Considering the potential outcome framework [41], we denote by $T^{a}$ the time to the event of interest that would have been observed had subjects been assigned to the exposure level a. Using this notation, we aim to quantify the causal effect of statin persistence by estimating the counterfactual survival curve, the average treatment effect at time t and the parameters of a marginal structural model (MSM), which are, respectively

(1) $$\begin{array}{ll} S^{a}(t) & =P(T^{a}>t) \end{array}$$

(2) $$\begin{array}{ll} ATE(t) & =S^{1}(t)-S^{0}(t) \end{array}$$

(3) $$\begin{array}{ll} \text{logit}[\lambda_{a}(t)] & =X_{a,t}^{⊤}\gamma \end{array}$$

where the counterfactual hazard is defined as

(4) $$\begin{array}{l} \lambda_{a}(t)=\underset{\Delta t\to 0}{\lim}P(t\leq T^{a}<t+\Delta t|T^{a}\geq t)/\Delta t \end{array}$$

and where $X_{a,t}^{⊤}$ is a row‐vector comprised of terms for the exposure a and time t. For example, $X_{a,t}^{⊤}$ could be (1,a,t,a×t) if the MSM of interest is $\text{logit}[\lambda_{a}(t)]=\gamma_{0}+\gamma_{1}a+\gamma_{2}t+\gamma_{3}a\times t$. The counterfactual survival curve $S^{a}(t)$ represents the proportion of people in the population of interest who would have remained event‐free at time t had everyone received exposure level A=a. The average treatment effect at time t is the difference between the proportion of event‐free individuals at time t had everyone been exposed (A=1) and had no one been exposed (A=0). Finally, the counterfactual hazard $\lambda_{a}(t)$ represents the incidence rate of the event at time t in a hypothetical population where the exposure would be A=a for everyone. Note that if the hazard is relatively close to 0 (for example $\lambda_{a}(t)<0.1$), then exp(γ) in the MSM can be interpreted as a hazard ratio. Otherwise, exp(γ) should be interpreted as an odds ratio. We also note that it can seem incorrect to model a hazard using a logit link since it is not a probability. However, as will be seen shortly, the follow‐up time will be discretized in practice and the hazard will thus be expressed as a (conditional) probability.

**FIGURE 1:**
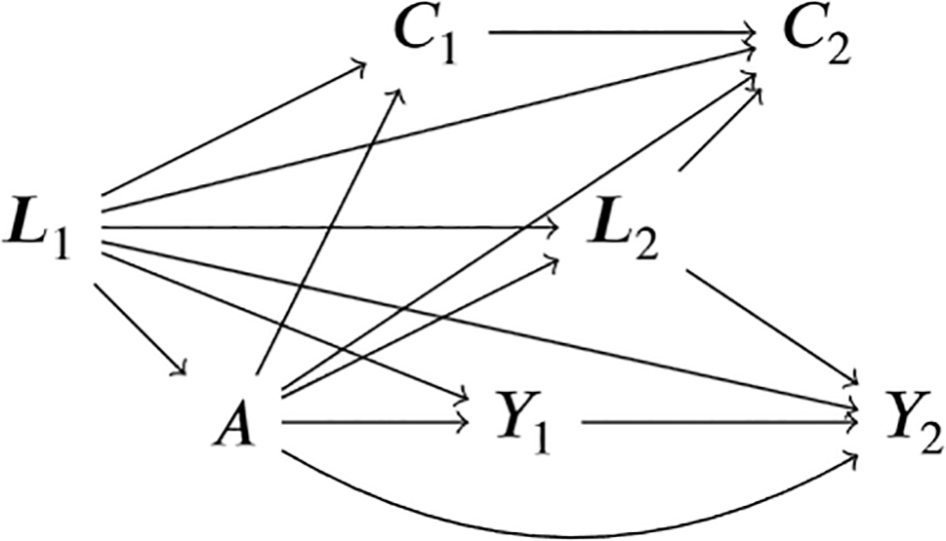
Directed acyclic graph representing the assumed causal structure of the data.

To estimate these quantities using the TMLE framework presented in this tutorial, the period of follow‐up must first be divided (discretized) into several relatively short sub‐periods of time (guidelines on how to perform this step are available in Section 5.3). Note that alternative TMLE algorithms that do not require discretizing follow‐up time have recently been introduced [42, 43, 44]. We will denote by t=1,…,K these time periods. As will be seen in Section 6, each individual is followed up to a maximum of two years after their statin initiation in our example. We could then, for example, divide the follow‐up period in sub‐periods of two months each, thus having t=1,…,12 periods of follow‐up. For each sub‐period, $Y_{t}=1$ denotes that the event of interest has occurred at time t or before time t and $Y_{t}=0$ indicates that the event has not yet occurred at time t (as such, whenever $Y_{t}=1$, then $Y_{t^{\prime }}=1$ for all $t^{\prime }>t$). A common feature of time‐to‐event outcomes is censoring, where the follow‐up of an individual ceases before the occurrence of the event. In our example, censoring can be due to loss to follow‐up (people exiting the study) or administrative end of follow‐up (end of the study period). Using notation similar to the one we used for the outcome, we will denote by $C_{t}=1$ censoring at or before time t and $C_{t}=0$ the absence of censoring (that is, the individual is still under follow‐up at time t). Once the individual is censored, their event indicator becomes missing, that is, if $C_{t}=1$, then $Y_{t},\ldots ,Y_{K}$ are missing. We denote by $L_{t}$ the covariates measured at time t, where we use $\ell_{t}$ to represent a corresponding data realization. In our example, these notably include age, sex, diabetes, and other risk factors of cardiovascular events. Finally, we denote by i=1,…,n the individuals that are part of the study. These subjects are assumed to be independently sampled from a given population. Figure 1 represents the assumed causal structure of the data using a directed acyclic graph in an example with K=2 time points for ease of representation. Note the assumed temporal ordering of the variables: $\left\{L_{1},A,C_{1},Y_{1},L_{2},C_{2},Y_{2},\ldots ,L_{K},C_{K},Y_{K}\right\}$. Notably, we allow for censoring between the exposure at baseline and the first outcome measurement at t=1. Alternative temporal orderings could be considered, which would require slight alterations to the implementation presented herein.

Identification of the causal exposure effect on the time‐to‐event outcome using observational data requires a few causal assumptions. These assumptions are not specific to TMLE; most estimators of such a causal effect require the same, or very similar, assumptions. First, there must be no unmeasured confounders, that is, the conditional exchangeability assumption; intuitively, there should be no unmeasured variable that affects both the exposure and the outcome, or the censoring and the outcome. To express this assumption more formally, we denote by $Y_{t}^{a,C_{t}=0}$ the outcome indicator that would have been observed at time point t under exposure A=a and under no censoring. This assumption is then expressed as $Y_{t}^{a,C_{t}=0}\;⊥\;⊥\;A|L_{1}$ and $Y_{t}^{a,C_{t}=0}\;⊥\;⊥\;C_{k}|A,{\bar{L}}_{k},C_{k-1}=0$ for all t and k≤t, where denotes statistical independence and ${\bar{L}}_{t}=(L_{1},L_{2},\ldots ,L_{t})$ is the history of the covariates up to time point t. A consistency assumption is also required. This assumption entails that the observed outcome at time point t of a given uncensored subject corresponds to the counterfactual outcome that would have been observed had the subject been assigned to their observed exposure, that is, A=a and $C_{t}=0\Rightarrow Y_{t}=Y_{t}^{a,C_{t}=0}$. Consistency could be violated if there are multiple versions of each exposure level [45]. A third assumption is the absence of interference, which means that the exposure of one subject does not affect the outcome of others. Finally, we must make positivity assumptions relative to the exposure and the censoring. For exposure, this requires that exposure assignment is not fully deterministic conditional on the observed baseline covariates $L_{1}$, that is, $0<P(A=1|L_{1}=\ell_{1})<1$ for all possible $\ell_{1}$. In other words, for all levels of the covariates' possible values, there should both be exposed and unexposed subjects in the population. For censoring, this requires that there are some uncensored individuals in the population at all time points in all strata of the previous covariates and exposure, that is, $P(C_{t}=0|A=a,{\bar{L}}_{t}=\bar{\ell_{t}})>0$, for all possible ($a,{\bar{\ell }}_{t}$).

### 3.1 Data Simulation

We now describe how we generated both counterfactual and observed simulated data compatible with a simplification of the general data structure given previously. The data were generated such that the previous causal assumptions are met. To simplify the illustration, we only consider K=4 time points and a single baseline covariate L. First we generated the covariate L as a binary variable with P(L=1)=0.5. Then the binary exposure variable A was generated as a function of L with P(A=1|L)=expit(−3+0.6L) where expit(x)=exp(x)/[1+exp(x)] is the inverse of the logit link function. We then generated the counterfactual probability of the event under exposure and under absence of exposure respectively at each time point t using the following equations

$$\begin{array}{ll} P(Y_{t}^{1}=1|Y_{t-1}^{1}=0,L) & =\text{expit}(-2-1+0.25L)\\ P(Y_{t}^{0}=1|Y_{t-1}^{0}=0,L) & =\text{expit}(-2+0+0.25L) \end{array}$$

For individuals that had already experienced the event at a previous time point, the event indicator remained fixed at 1 ($Y_{t}^{a}=1$ if $Y_{t-1}^{a}=1$). The observed outcome at each time point corresponded to the counterfactual outcome under the observed exposure (if A=1 then $Y_{t}=Y_{t}^{1}$, otherwise $Y_{t}=Y_{t}^{0}$). The censoring indicator at each time point was generated as a function of A and L as $P(C_{t}=1|C_{t-1}=0,A,L,Y_{t-1}=0)=\text{expit}(-5+0.2A+0.2L)$. Again, individuals that had been censored at a previous time point remained censored ($C_{t}=1$ if $C_{t-1}=1$) and all future event indicators were set to missing. R code for generating the data is available in Box 1.

Box 1 **Function to generate the data.**

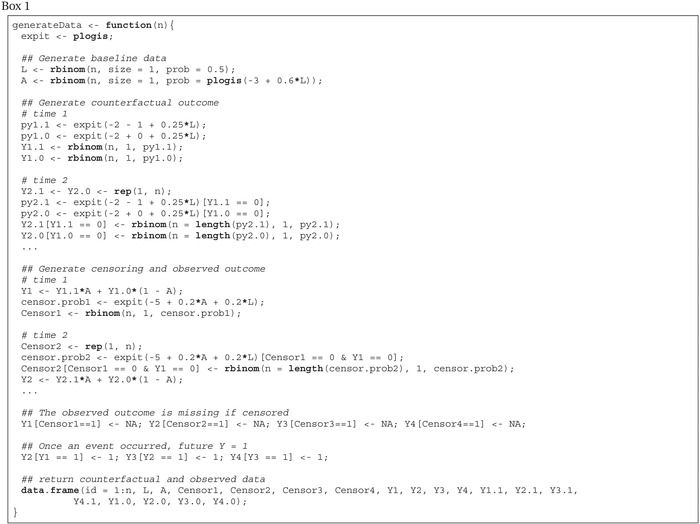

To determine the true causal effect, we generated 5 000 000 counterfactual observations and directly calculated the counterfactual survival probabilities in Equation (1) and found (rounding at the second decimal place) $S^{1}(1)=0.95$, $S^{1}(2)=0.90$, $S^{1}(3)=0.85$, $S^{1}(4)=0.80$, $S^{0}(1)=0.87$, $S^{0}(2)=0.75$, $S^{0}(3)=0.65$ and $S^{0}(4)=0.56$. Because the event can only occur at discrete time points in this simulated example, note that

(5) $$\begin{array}{l} \lambda_{a}(t)=P(T^{a}=t|T^{a}\geq t)=\frac{S^{a}(t-1)-S^{a}(t)}{S^{a}(t-1)} \end{array}$$

Using Equation (5), we computed the counterfactual hazards, for example $\lambda_{1}(3)=[S^{1}(2)-S^{1}(3)]/S^{1}(2)=[0.90-0.85]/0.85$. The next step was to choose a working MSM relating these hazards to exposure and time. We chose the MSM $\text{logit}[\lambda_{a}(t)]=\gamma_{0}+\gamma_{1}a+\gamma_{2}I(t=2)+\gamma_{3}I(t=3)+\gamma_{4}I(t=4)$, where I(·) is the usual indicator function that takes the value 1 when its argument is true and 0 otherwise. Following Schnitzer et al. (2014) [35], the parameters of this MSM were determined by fitting a weighted logistic regression of the counterfactual hazards ($\lambda_{a}(t)$) as the dependent variable on exposure and time as independent variables, with weights equal to $S^{a}(t-1)$. Recall from Equation (5) that the hazards are conditional probabilities when time has been discretized, thus supporting the use of a logistic regression. We obtained the following parameters: $\gamma_{0}=-1.87$, $\gamma_{1}=-1.00$, $\gamma_{2}=\gamma_{3}=\gamma_{4}=-0.01$. In this example, only $\gamma_{1}$ has a causal interpretation, since it is the only parameter related to the exposure. The other parameters account for the fact that the hazard may vary in time, though the exposure effect is constant over time. These other parameters will not be interpreted. Note that some hazards are not sufficiently close to 0 for $\exp(\gamma_{1})$ to be interpreted as the exposure hazard ratio. Instead, $\exp(\gamma_{1})$ should be interpreted as the exposure odds ratio.

In this simple example, the independent censoring assumption is met, since there are no post‐exposure variables affecting both the outcome and censoring indicators. However, the steps we describe in the tutorial below allow for such post‐exposure variables to exist.

## 4 Tutorial

Before moving to the step‐by‐step tutorial on how to use TMLE to estimate our causal quantities of interest (also called the target parameters), we first provide a brief overview of the general TMLE framework. TMLE first requires constructing and fitting a model for the outcome according to the exposure and confounders. The output of this outcome model fit can then be used to obtain a first estimate of the causal quantities of interest. In general, this is achieved by calculating the predicted value of the outcome under exposure and under no exposure for all individuals, in order to estimate the corresponding counterfactual outcome expectations. However, these estimates rely on the correct specification of the outcome model to be valid. In a second step, TMLE requires modeling the exposure according to the confounders. The information of this exposure model is then used to compute a weight that is used to update the initial estimate. The intuition is as follows: If the outcome model was correctly specified, then the exposure model should not provide any additional information for predicting the observed outcome beyond the initial outcome model. Indeed, both the outcome and the exposure models adjust for the same covariates. Because the same data cannot provide new information, the exposure model's predictions should be independent of the outcome model's errors. However, if the initial outcome model is incorrectly specified, then the exposure model predictions may improve the initial outcome model fit. As such, it provides a second occasion for correctly modeling the relation between the outcome and exposure and covariates. This is why TMLE benefits from the double robustness property we mentioned in the introduction. More technically, TMLE is constructed in such a way that the final estimator solves the estimating equations of the efficient influence curve of the target parameter. The influence curve of an estimator can be conceptualized as its core component, which determines its large‐sample (asymptotic) properties. The efficient influence curve is optimal for a given target parameter in the sense that an estimator that solves the corresponding estimating equations has the lowest possible asymptotic variance for the target parameter in its class of estimators, assuming that the outcome and exposure models were correctly specified (local efficiency). The variance of a TMLE estimator can be estimated by computing the sample variance of the empirical version of the efficient influence curve, scaled by a factor 1/n. A more detailed presentation of the TMLE framework can be found elsewhere [1, 37]. Note that the updating step was traditionally performed using a “clever covariate”, corresponding to the IPTW, which was included as the sole covariate in an (unweighted) intercept‐free regression model for the observed outcome and with an offset equal to the initial predicted outcome (appropriately scaled). The weighted‐regression approach we instead describe has been observed to have better finite‐sample performance [8].

We now provide a detailed, step‐by‐step tutorial on how to implement TMLE to estimate the counterfactual survival curves and the MSM for the outcome hazard in the simple simulation with observed data structure $O=(L,A,C_{1},Y_{1},C_{2},Y_{2},C_{3},Y_{3},C_{4},Y_{4})$. We provide R code for each step. As will be seen, there are some important and particular aspects of implementing TMLE when modeling a censored time‐to‐event outcome. First, for the algorithm we present, the event indicator at each time point must be modeled. Moreover, the censoring mechanism must also be modeled. The algorithm also requires recursive operations to estimate the survival probabilities at later time points. As mentioned previously, the tutorial makes use of simulated data to increase replicability. These data were obtained by generating n=5000 observations using the function in Box 1 setting the seed for the random number generator to 1234 and discarding the counterfactual outcomes.

### 4.1 Estimating $S^{a}(1)$

We begin by estimating the counterfactual survival probability at the first time point. The R code associated with the steps below is provided in Box 2.

#### 4.1.1 Step 1—Modeling the Outcome at the First Time Point

In order to obtain initial estimates of $S^{1}(1)$ and $S^{0}(1)$, we first model the event indicator at the first time point ($Y_{1}$) as a function of exposure A and baseline covariate L among individuals who were uncensored at the first time point ($C_{1}=0$). We correspondingly define the conditional event probability $Q^{A}(1,L)=P(Y_{1}=1|A,L,C_{1}=0)$. In this example, we used the logistic regression model $\text{logit}[Q^{A}(1,L)]=\beta_{0}+\beta_{1}A+\beta_{2}L$ to simplify the illustration, but using machine learning algorithms would be preferable to avoid assuming a parametric model. The use of machine learning within TMLE is further discussed in Section 5.4. Next $S^{a}(1)=P(Y_{1}^{a}=0)=1-P(Y_{1}^{a}=1)$ can be estimated by computing one minus the average of the model's predicted values over all subjects (censored or uncensored), fixing the exposure level to A=a. For example,

$$\begin{array}{ll} {Ŝ}^{1}(1) & =1-\frac{1}{n}\overset{n}{\underset{i=1}{\sum }}\hat{P}(Y_{1}=1|A=1,L=\ell_{i})\\ & =1-\frac{1}{n}\overset{n}{\underset{i=1}{\sum }}\text{expit}({\hat{\beta }}_{0}+{\hat{\beta }}_{1}+{\hat{\beta }}_{2}\ell_{i}) \end{array}$$

Intuitively, this step can be seen as predicting what would have been the probability of not having an event at the first time point for all individuals, had they been exposed and uncensored, possibly contrary to the fact. In addition to the causal assumptions laid out previously, this estimator also requires the correct specification of the outcome (event) model at the first time point to be valid. For convenience, we will denote by ${\hat{Q}}^{a}(1,\ell_{i})=\hat{P}(Y_{1}=1|A=a,L=\ell_{i})$ the individual predicted event probabilities.

#### 4.1.2 Step 2—Modeling the Exposure A and Censoring $C_{1}$ at the First Time Point

As a preparatory step for updating the initial estimate we obtained in step 1, we need to model the exposure conditional on baseline covariates and censoring at the first time point conditional on exposure and baseline covariates. Again, while using machine learning algorithms would be preferable, we used the logistic regression models $\text{logit}[P(A=1|L)]=\alpha_{0}+\alpha_{1}L$ and $\text{logit}[P(C_{1}=1|A,L)]=\delta_{0}+\delta_{1}A+\delta_{2}L$ in this example to simplify the illustration.

#### 4.1.3 Step 3—Update the Initial Estimate ${\hat{Q}}^{a}(1,\ell_{i})$

To update the initial estimate ${\hat{Q}}^{1}(1,\ell_{i})$ we first need to compute the inverse probability weight ${Ĥ}_{1}(1,\ell_{i})$ for all subjects:

$$\begin{array}{l} {Ĥ}_{1}(1,\ell_{i})=\frac{I(A=1,C_{1}=0)}{\hat{P}(A=1|L=\ell_{i})\hat{P}(C_{1}=0|A,L=\ell_{i})} \end{array}$$

using the predicted probabilities of censoring and exposure. This inverse probability weight is then used to determine how the initial estimate ${\hat{Q}}^{a}(1,\ell_{i})$ should be updated. Recall that this inverse probability weight is used to update the initial estimator so that the resulting estimator solves the estimating equations of the efficient influence curve and thus inherits asymptotic local efficiency and double robustness. This is achieved by running the following logistic regression with weights ${Ĥ}_{1}(1,L)$ among subjects that were uncensored at time 1:

$$\begin{array}{l} \text{logit}[P(Y_{1}=1|C_{1}=0,L)]=\text{logit}\left[{\hat{Q}}^{1}(1,L)\right]+\varepsilon_{1}(1) \end{array}$$

This corresponds to a weighted logistic regression of the observed event indicator at time point one with only an intercept $\varepsilon_{1}(1)$ and the logit of the individual initial estimates ${\hat{Q}}^{1}(1,\ell_{i})$ as an offset term. The estimated intercept ${\hat{\varepsilon }}_{1}(1)$ can informally be conceptualized as a measure of the residual association between the observed outcome and the covariate L. Each individual estimate ${\hat{Q}}^{1}(1,\ell_{i})$ can then be updated using the following formula

$$\begin{array}{l} {\hat{Q}}^{1,*}(1,\ell_{i})=\text{expit}\left\{\text{logit}\left[{\hat{Q}}^{1}(1,\ell_{i})\right]+{\hat{\varepsilon }}_{1}(1)\right\} \end{array}$$

Finally, the updated survival estimate ${Ŝ}^{1,*}(1)$ is obtained by computing the average of the individual updated predictions $1-{\hat{Q}}^{1,*}(1,\ell_{i})$. When implemented with parametric estimators for the nuisance quantities as demonstrated here, the updated estimate ${Ŝ}^{1,*}(1)$ is doubly‐robust: It is valid if either the outcome model in Step 1 is correctly specified, or if the exposure and the censoring models in Step 2 are correctly specified, but the three models do not all need to be correctly specified. As such, the statistical assumptions required to obtain a valid estimate are less stringent than those of the initial estimate. However, the causal assumptions remain the same.

Similarly, updating the initial estimate ${Ŝ}^{0}(1)$ requires computing

$$\begin{array}{l} {Ĥ}_{0}(1,\ell_{i})=\frac{I(A=0,C_{1}=0)}{\hat{P}(A=0|L=\ell_{i})\hat{P}(C_{1}=0|A,L=\ell_{i})} \end{array}$$

then running the logistic regression $\text{logit}[P(Y_{1}=1|C_{1}=0,L)]=\text{logit}\left[{\hat{Q}}^{0}(1,L)\right]+\varepsilon_{0}(1)$, weighted by ${Ĥ}_{0}(1,L)$, and computing ${\hat{Q}}^{0,*}(1,\ell_{i})=\text{expit}\left\{\text{logit}\left[{\hat{Q}}^{0}(1,\ell_{i})\right]+{\hat{\varepsilon }}_{0}(1)\right\}$ and finally computing the average of $1-{\hat{Q}}^{0,*}(1,\ell_{i})$.

#### 4.1.4 Step 4—Estimate the Variance of ${ŝ}^{a}(1)$

The efficient influence curve of ${Ŝ}^{a}(1)$ is given by [35]

(6) $$\begin{array}{l} IC_{a}(1)=H_{a}(1,L)\left(Q^{a,*}(1,L)-Y_{1}\right)+S^{a,*}(1)-Q^{a,*}(1,L) \end{array}$$

A simple variance estimator for ${Ŝ}^{a}(1)$ is obtained by computing the sample variance of Equation (6) (with estimates of each quantity) scaled by a factor 1/n. A 95% Wald confidence interval is then obtained by computing ${Ŝ}^{a,*}(1)\pm z_{0.975}\sqrt{\hat{\mathrm{Var}}\left[{Ŝ}^{a,*}(1)\right]}$. The efficient influence curve for the average treatment effect at time 1 (ATE(1)) is the difference in the efficient influence curves of ${Ŝ}^{1}(1)$ and ${Ŝ}^{0}(1)$.

Following these steps, the estimates (95% confidence intervals [CI]) were ${Ŝ}^{1}(1)=0.94$ (0.91,0.97), ${Ŝ}^{0}(1)=0.87$ (0.86,0.88), and $\hat{AT\;E}(1)=0.07$ (0.04,0.10).

Box 2 **R code for estimating S^a^(1).**

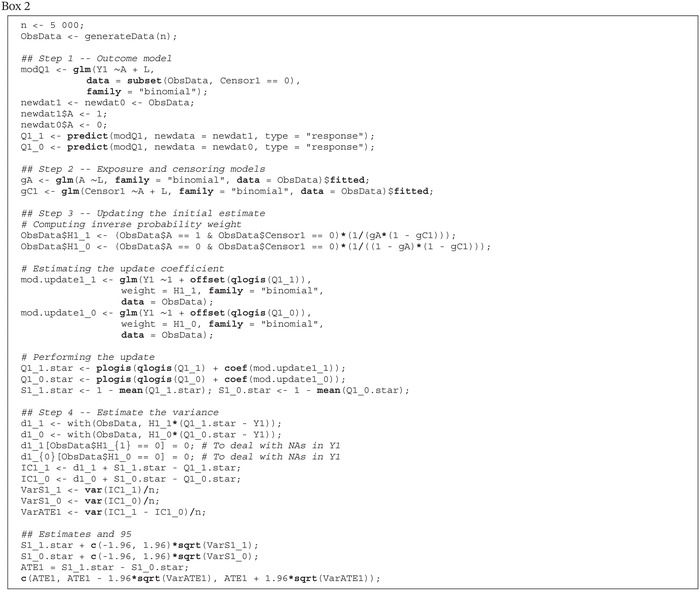

### 4.2 Estimating $S^{a}(t)$ for t≥2

We now provide the general steps for estimating the counterfactual survival probabilities at the other time points. Because most of these steps are similar to those we have just seen, we focus on the differences. Recall that the complete R code is available on GitHub (https://github.com/detal9/SurvTMLE).

Algorithm 1 describes the general algorithm for the estimation of $S^{a}(t)$ using steps similar to Steps 1–4 of Section 4.1. However, note that the order in which we present the steps differs. In Step 1a, a model for the exposure as a function of baseline covariates is fitted. In Step 1b, a model is fitted for the censoring at each time point j=1,…,t among individuals that were uncensored at time j−1 and who did not have the event yet at j−1, as a function of exposure and covariates up to time j. The rest of the algorithm proceeds recursively by first setting j=t. The second step (2a) is to model the event indicator at time j among subjects who were uncensored at time j and who did not have the event yet as a function of exposure and covariates up to time j. Then predicted event probabilities under A=a are computed for all individuals who were uncensored at time j−1 (2b). For those who had the event at time j−1, the predicted probability is set to 1 since $Y_{j}=1$ when $Y_{j-1}=1$. In Step 3, the initial estimate of the event probability obtained in Step 2 is updated. Step 4 computes a component of the variance of ${Ŝ}^{a}(t)$. Steps 2–4 are then repeated for j=t−1,t−2,…,1, replacing the observed outcome indicator of Step 2 by the final updated estimate of the event probability obtained in the previous Step 4. Intuitively, this algorithm that moves backward in time allows for the estimation of the event probability at time t for subjects who were uncensored as well as those that were censored at time t, then additionally among those censored at time t−1, and so forth. We provide a more formal explanation of this algorithm based on iterated nested expectations in the Appendix A.

Following the previous steps, we obtained the estimates (95% CI): ${Ŝ}^{1}(2)=0.88$ (0.84,0.92), ${Ŝ}^{0}(2)=0.75$ (0.74,0.76), ${Ŝ}^{1}(3)=0.83$ (0.79,0.88), ${Ŝ}^{0}(3)=0.65$ (0.64,0.67), ${Ŝ}^{1}(4)=0.79$ (0.75,0.84), ${Ŝ}^{0}(4)=0.57$ (0.55,0.58). As can be seen, the estimates we obtained here are very close to the true values reported in Section 3. The estimated average treatment effects are $\hat{AT\;E}(2)=0.13$ (0.09,0.17), $\hat{AT\;E}(3)=0.18$ (0.13,0.22), and $\hat{AT\;E}(4)=0.23$ (0.18,0.28).

ALGORITHM 1 **General TMLE algorithm for estimating $S^{a}(t)$ with observed survival data structure $O=(L_{1},A,C_{1},Y_{1},L_{2}\ldots ,L_{K},C_{K},Y_{K})$.**

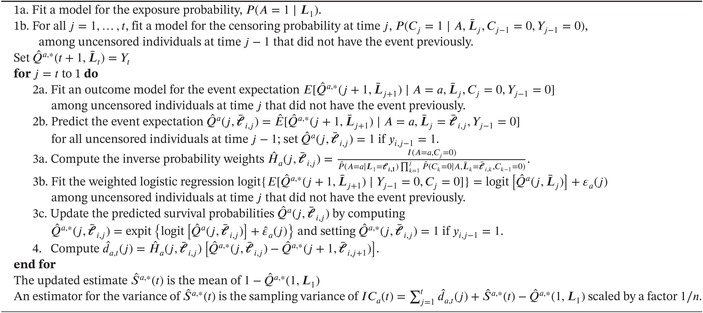

### 4.3 Modeling the Hazard

Once the counterfactual survival probabilities $S^{a}(t)$ have been estimated for all t=1,…,K, the counterfactual hazards $\lambda_{a}(t)$ can be estimated. This is achieved by inserting the estimated counterfactual survival probabilities in Equation (5). To model these hazards as a function of exposure and time, the next step is to fit a model $\text{logit}[{\hat{\lambda }}_{a}(t)]=X_{a,t}^{⊤}\gamma$ where the outcomes are the 2K estimated hazards and X is a design matrix with 2K rows corresponding to each $X_{a,t}^{⊤}$, weighting each observation (each hazard) according to ${Ŝ}^{a}(t-1)$. In our example, we fit the model $\text{logit}[{\hat{\lambda }}_{a}(t)]=\gamma_{0}+\gamma_{1}a+\gamma_{2}I(t=2)+\gamma_{3}I(t=3)+\gamma_{4}I(t=4)$ using data with the following structure, where the columns of X are (1,a,I(t=2),I(t=3),I(t=4))

$$\begin{array}{ll} {\hat{\lambda }}_{a}(t) & =\left(\begin{array}{c} {\hat{\lambda }}_{1}(1)\\ {\hat{\lambda }}_{0}(1)\\ {\hat{\lambda }}_{1}(2)\\ {\hat{\lambda }}_{0}(2)\\ {\hat{\lambda }}_{1}(3)\\ {\hat{\lambda }}_{0}(3)\\ {\hat{\lambda }}_{1}(4)\\ {\hat{\lambda }}_{0}(4) \end{array}\right),\;X=\left(\begin{array}{ccccc} 1 & 1 & 0 & 0 & 0\\ 1 & 0 & 0 & 0 & 0\\ 1 & 1 & 1 & 0 & 0\\ 1 & 0 & 1 & 0 & 0\\ 1 & 1 & 0 & 1 & 0\\ 1 & 0 & 0 & 1 & 0\\ 1 & 1 & 0 & 0 & 1\\ 1 & 0 & 0 & 0 & 1 \end{array}\right)\;\\ \text{weights} & =\left(\begin{array}{c} 1\\ 1\\ {Ŝ}^{1}(1)\\ {Ŝ}^{0}(1)\\ {Ŝ}^{1}(2)\\ {Ŝ}^{0}(2)\\ {Ŝ}^{1}(3)\\ {Ŝ}^{0}(3) \end{array}\right) \end{array}$$

Because this regression is a function of the estimated counterfactual survival probabilities ${Ŝ}^{a}(t)$, the variance of the regression coefficient can be computed as a function of the variance of ${Ŝ}^{a}(t)$. More precisely, a variance estimator for the regression coefficients is obtained using the functional Delta‐method (see Zepeda‐Tello et al., 2022 [46] for a tutorial on this topic). Following Schnitzer et al. (2014) [35] (also see Appendix B), the efficient influence curve of γ is

$$\begin{array}{ll} IC_{\gamma } & ={\left\{\underset{a,t}{\sum }S_{a}(t-1)\frac{\exp(X_{a,t}^{⊤}\gamma )}{{\left[1+\exp(X_{a,t}^{⊤}\gamma )\right]}^{2}}X_{a,t}X_{a,t}^{⊤}\right\}}^{-1}\\ & \;\times \underset{a,t}{\sum }\left\{-X_{a,t}+X_{a,t+1}{\left[1+\exp(X_{a,t+1}^{⊤}\gamma )\right]}^{-1}\right\}IC_{a}(t) \end{array}$$

$IC_{\gamma }$ is a matrix with n rows and as many columns as there are γ coefficients. An estimator of the variance‐covariance matrix of $\hat{\gamma }$ is obtained by computing the sample variance of the empirical version of $IC_{\gamma }$, scaled by a factor 1/n. This formula is obtained by treating the data as n independent replications of the longitudinal sequence $\left\{L_{1},A,C_{1},Y_{1},L_{2},C_{2},Y_{2},\ldots ,L_{K},C_{K},Y_{K}\right\}$. It does not require assuming that there is no within‐patient correlation, or assuming a specific structure for the within‐patient correlation. Box 3 provides the R code associated with those steps. In our example, this yields the following estimates (95%CI): ${\hat{\gamma }}_{0}=-1.86$ (−2.02,−1.71), ${\hat{\gamma }}_{1}=-0.93$ (−1.22,−0.64), ${\hat{\gamma }}_{2}=0.04$ (−0.23,0.31), ${\hat{\gamma }}_{3}=-0.05$ (−0.32,0.23), ${\hat{\gamma }}_{4}=-0.08$ (−0.39,0.22). Again, these estimates are very close to the true values we computed in Section 3.

Box 3 **R code for estimating the hazard model.**

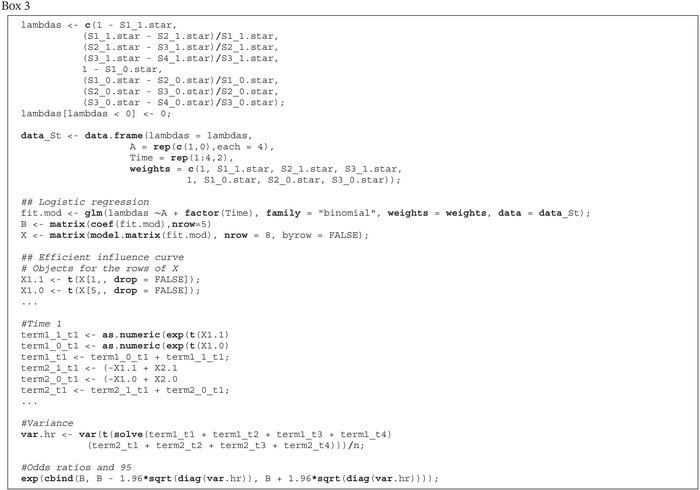

## 5 Guidelines

We now provide recommendations on how best to use TMLE to estimate the effect of a point exposure on a time‐to‐event outcome.

### 5.1 Type of Research Questions

The method we have presented in this tutorial is only appropriate for estimating the effect of a binary or categorical point exposure on a time‐to‐event outcome in the presence of missing‐at‐random censoring. This framework is suitable for many common research questions provided that machine learning methods are used to avoid making inappropriate parametric assumptions. For example, this approach can be used to estimate the effect of starting on Drug A versus starting on Drug B among new users. As another example, the TMLE we have presented could also be used to compare the time to recurrence or death of people who survived a first myocardial infarction and returned to work within a month according to whether they return to work full‐time or part‐time. Research questions that involve a time‐varying exposure, such as per protocol analyses or estimating the effect of a cumulative exposure, cannot be answered with the specific TMLE we have described herein. However, extensions allowing for time‐varying exposures are available and described elsewhere [35, 40]. Extensions of TMLE for time‐to‐event outcomes that accommodate continuous exposures are not immediately available as dose‐response estimands are not pathwise‐differentiable parameters [47], though other estimators for specific contexts are sometimes discussed (e.g., [48]).

### 5.2 Appropriate Study Design and Variables to Measure

As in all studies considering a time‐to‐event outcome, the design must comprise some longitudinal aspect. To be able to adequately control the potential selection bias due to censoring, one must measure not only baseline confounders, but also time‐varying covariates that are susceptible to affect both censoring and outcome. This does not constitute a weakness of TMLE as compared to traditional approaches such as Cox regression, but rather a strength, since it allows TMLE to overcome the commonly made independent censoring assumption. We note that baseline confounders, by definition and assumption, must not have been affected by the exposure. As such, it may be preferable that baseline covariates be measured some time before the exposure. Using a causal diagram, constructed using the literature and expert knowledge, can be helpful in identifying which variables to measure and include in the analysis [49, 50].

Regarding the choice of variables to adjust for, it can be noted in Algorithm 1 that both the model for the expected outcome at time j, $E({\hat{Q}}^{a,*}(j+1)|A,{\bar{L}}_{j},C_{j}=0,Y_{j-1}=0)$, and the model for the probability of censoring at time j, $P(C_{j}=1|A,{\bar{L}}_{j},C_{j-1}=0,Y_{j-1}=0)$, formally depend on all previous covariates ${\bar{L}}_{j}=(L_{1},L_{2},\ldots ,L_{j})$. It might not be feasible to fit models conditional on the complete covariate history in practice, especially in applications with numerous time points or a small sample. Fortunately, variables that can be assumed not to be predictive of the outcome at time j can be excluded from the models. For example, if one is willing to assume that the event risk only depends on the exposure and the covariates measured at the two most recent time points, one could correspondingly fit a model for $P(C_{j}=1|A,L_{j},L_{j-1},C_{j-1}=0,Y_{j-1}=0)$.

### 5.3 Discretization of the Follow‐Up Period

As previously mentioned, the follow‐up period must be discretized in several sub‐periods when using the TMLE algorithm we have presented. The choice of the length of the sub‐periods can be challenging in practice. Selecting shorter periods of time avoids a loss of information, but can lead to data‐sparsity [51]. We thus recommend dividing the follow‐up into relatively short sub‐periods of time while ensuring that some outcome events and censoring occur in each sub‐period. Note that it is not strictly necessary for all sub‐periods to have the same length. For example, longer sub‐periods could be used when fewer events occur and shorter sub‐periods could be used when there are more events.

### 5.4 Using Machine Learning in TMLE

When estimating $S^{a}(t)$ using TMLE, there are three types of models that need to be fitted: A model for the exposure probability, models for the censoring indicators, and models for the event indicators. In the tutorial we have presented above, we used logistic regression models for each of these. However, as mentioned in the introduction, one key advantage of using TMLE is that it allows for the usage of data‐adaptive procedures (machine learning) to fit its component models. While the usage of such procedures does not protect against residual confounding due to unmeasured confounders, it can minimize residual confounding due to incorrect modeling of associations with measured confounders. Indeed, using machine learning algorithms allows the model to learn from the data how best to model associations with confounders. To estimate $S^{a}(t)$ using TMLE together with machine learning algorithms, one only needs to replace the logistic regression models we have used in Steps 1 and 2 of the algorithms with appropriate machine learning approaches. Nothing else in the algorithm changes. In addition, because the MSM for the hazards is a function of the estimated ${Ŝ}^{a}(t)$, this part of the approach also remains unchanged. For modeling the exposure and censoring, methods for binary or multi‐level dependent variables should be used, whereas methods appropriate for modeling bounded continuous probabilities should be used to model the outcome.

Even though TMLE better combines with machine learning than singly‐robust approaches, care must be given to the choice of algorithms to use [52]. Indeed, it is important to choose approaches that converge fast enough to the true model as sample size increases, and that are additionally not too adaptive (i.e., they meet the Donsker condition) to guarantee the statistical properties of TMLE. Tree‐based methods, such as random forests and gradient boosting, have been observed to yield biased estimates and confidence intervals that include the true parameters less often than they should (i.e., undercoverage) in simulation studies [32]. As such, some authors have recommended using less flexible methods like generalized additive models or multivariate adaptive regression splines [52]. It is very common to use Super Learner [53] together with TMLE. Super Learner is an ensemble method that produces predicted values as a linear combination of the predictions of multiple methods (the learners), where the weight attributed to each learner is determined using cross‐validation [53]. An advantage of Super Learner is that it is expected to put large weights on simpler algorithms in its library whenever they are appropriate. As such, it is recommended to include several methods of diverse complexity in the Super Learner library [52]. If it is believed that the flexibility of tree‐based methods is required to adequately control for confounding, then some form of sample‐splitting should be used because it circumvents the need for the Donsker condition [52, 54]. Cross‐fitting is a particularly interesting type of sample‐splitting procedure because of how efficiently it uses the data. A review of cross‐fit estimators for causal inference can be found elsewhere [55]. The highly adaptive lasso is a very flexible algorithm that meets the theoretical conditions (i.e., adequate convergence rate and Donsker condition) required to be used without cross‐fitting [56, 57]. Guidelines concerning the use of the Super Learner have recently been published [58]. These guidelines notably provide guidance in determining the number of cross‐validation splits and which learners to include in the Super Learner.

### 5.5 Choice of Estimand

We have demonstrated how to estimate counterfactual survival probabilities and counterfactual hazards. As indicated earlier, hazards may sometimes require stronger assumptions to be interpreted causally compared to risks. This is because the discrete‐time hazard conditions on survival up to time t−1. If there are unmeasured common causes of survival status at t and t−1 we typically get selection bias due to paths such as A→Yt−1←U→Yt. This means that the hazards at time t may differ between the two exposure groups simply because of different individuals who survive until t−1 under a=1 versus a=0—because of exposure effects before t−1 [59]. This suggests that we should ideally always calculate the counterfactual survival probabilities, though the counterfactual hazards may give us a better direct comparison to regression estimates [16].

### 5.6 R Function

We developed an R function, surv.TMLE that is available on GitHub (https://github.com/detal9/SurvTMLE) to implement TMLE, which we briefly describe herein. The complete documentation and examples using simulated data are available on GitHub. To use this function, the data must first be arranged in a wide format, with a single row for each individual. The Yvar, Cvar, Avar, Lvar, L0var arguments are used to supply the names of the outcome ($Y_{1}$, …, $Y_{K}$), censoring ($C_{1}$, …, $C_{K}$), exposure (A), time‐varying confounders ($L_{1}$, …, $L_{K}$) and time‐fixed baseline variables, respectively. All models are adjusted for time‐fixed baseline variables, whereas it is possible to choose using “lookback” arguments how many previous time‐points should be used when modeling the outcome and censoring at each time‐point (recall the discussion in the second paragraph of Section 5.2). The Ymod, Cmod, Amod arguments are used to determine if parametric models (=“parametric”) or machine learning (=“SL”) methods should be used to model the outcome, the censoring and the exposure, respectively. If machine learning methods are used, SL.library arguments are available to specify which learners should be considered. Note that with a multilevel categorical exposure, A.SL.library is ignored when Amod = “SL”, and a polychotomous regression and multiple classification algorithm is used [60], since the SuperLearner package in R does not currently accommodate multinomial dependent variables. The argument MSM.form can optionally be used to specify the right hand side of a formula for an MSM relating the logit of the hazards to exposure and time. The gbound argument is used as an ad‐hoc solution to near‐positivity violations. It is used to replace estimated value of exposure and censoring probabilities that are too close to zero by the value set in gbound. Following recent guidelines [58], the default behavior of the function is to adapt the number of cross‐validation folds of each Super Learner according to the effective sample size. Our function uses the mean squared error as a loss function within Super Learner, although recent guidelines recommend using the mean negative log‐likelihood when modeling binary data [58]. We tried using the latter loss function, but this resulted in errors in examples with larger sample sizes.

## 6 Application

### 6.1 Data

We now consider a real data illustration concerned with the estimation of the effect of statin persistence for at least three months after initiation on the occurrence of a first cardiovascular event or death, among adults aged 66 or older in Quebec, Canada.

We used medical administrative data from the Quebec Integrated Chronic Disease Surveillance System, which is formed by the linkage of five administrative databases: The health insurance registry, the pharmaceutical services database, the physician claims database, the hospitalization database, and the death registry [61]. Quebec has a public health insurance plan to which it is mandatory to subscribe. In addition, the vast majority (>90%) of people aged 65 and older are covered by the public drug insurance plan. We included people aged 66 or older who claimed a statin treatment between 2008 and 2013, who had no claim for statins in the previous year, who had no history of cardiovascular disease in the five years prior to their first claim of statins and who did not live in a long‐term care residence in the previous year (because data on drug claims are unavailable for them). A total of 69 632 people were thus included. Statin treatment was identified using common denomination codes corresponding to atorvastatin, fluvastatin, lovastatin, pravastatin, rosuvastatin, simvastatin, and the combination niacin/lovastatin. Cardiovascular diseases were identified using validated definitions [62, 63] and mostly consisted of strokes, transient ischemic attacks, myocardial infarction, unstable angina, coronary artery bypass graft and percutaneous coronary interventions.

The exposed group (A=1) comprised people for whom there was no gap in refilling their statin claim over the three months following initiation, after considering a 50% grace period and any additional hospitalization periods (because drugs are supplied by the hospital during these periods). In Quebec, these drugs are often supplied for a duration of 30 days, or of 7 days for people using a blister card. The non‐exposed group (A=0) comprised people who had at least one gap in the same time‐period. The follow‐up for events started after this three‐month period. We excluded 3500 people (5%) at this stage because they experienced a first cardiovascular event, died or were transferred to a long‐term residence during the three‐month period used to assess exposure. The remaining 66 132 people were followed until the occurrence of a first cardiovascular event or death from any cause (Y=1), discontinuation of their subscription to the public health insurance plan (e.g., moving out of the province, C=1) or for a maximum of two years (C=1).

The following covariates were considered as potential confounders based on experts' knowledge: Age, sex (male/female), residence area (urban = ≥ 10 000 inhabitants/rural), number of visits in the year prior to statin initiation to a generalist, to a specialist, to an emergency room, total number of hospitalizations in the year prior to statin initiation, prevalent diabetes (yes/no), hypertension (yes/no), chronic kidney disease (yes/non), a comorbidity score combining Charlson and Elixhauser scores [64], number of different medications claimed in the year prior to statin initiation, a material and social deprivation index (where missing values are considered as a category) [65], aspirin or antiplatelet agents claims (yes or no), oral anticoagulants (yes/no), blood pressure therapy (yes/no), and other lipid‐lowering drugs (yes/no). Only baseline values of these variables were considered.

The project was approved by the ethics board of the CHU de Québec (# 2020–4892).

### 6.2 Analysis

Two different approaches were used to estimate the effect of statin persistence on the occurrence of a cardiovascular event or death. First, we used a Cox model adjusted only for baseline covariates. Next, we used the TMLE approach described in this tutorial, with follow‐up divided into 12 2‐month periods. We chose to consider two‐month periods to avoid data sparsity. In the TMLE procedure, the exposure, censoring and outcome processes were estimated using a Super Learner with a simple mean, main terms logistic regression, a logistic regression with two‐way interaction terms and quadratic terms for continuous variables, a generalized additive model, and earth (an implementation of multivariate adaptive regression splines) as learners. These learners were chosen to try to include diverse levels of flexibility among the learners, while excluding highly flexible approaches, which would require sample splitting to be valid [52]. A relatively small library was chosen to keep computational burden reasonable (three hours to run the TMLE analysis). We tried to include a modified lasso that accommodates bounded continuous outcomes, both as a possible learner and a screener for pre‐selecting covariates, but this resulted in errors. The highly adaptive lasso could not be used due to installation restrictions of the server on which analyses were conducted. In our application, 169 Super Learners were fitted: One for the exposure, 12 for censoring probabilities at each time point, and 154 for the outcome (2 exposure levels × 12 time points × t per time point). The number of cross‐validation folds was determined data‐adaptively and was two for modeling the exposure, and between two and ten for modeling the censoring and outcome at different time‐points. Box 4 provides the R code used to implement TMLE with Super Learner in our illustration.

Box 4 **R code for the real data illustration.**

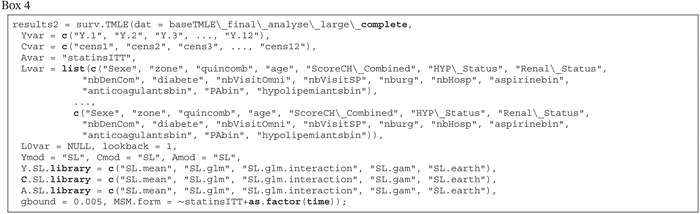

### 6.3 Results

Table 2 describes the baseline characteristics of the sample. Most people remained persistent for at least three months (93.6%). As compared to non‐persistent users, persistent users were more likely to have hypertension, and to claim aspirin and blood pressure treatment. Otherwise, non‐persistent and persistent users had similar baseline characteristics.

**TABLE 2:** Baseline characteristics of new statin users aged 66 or more without history of cardiovascular disease according to three‐month persistence status in Quebec, Canada.

	Persistent	Non‐persistent
Variables	n = 61 890 (93.6%)	n = 4242 (6.4%)
Sex, *n* (%)		
Female	33 981 (54.9)	2530 (59.6)
Male	27 909 (45.1)	1712 (40.4)
Age, mean (SD)	71.1 (5.6)	71.0 (6.0)
Residence area, *n* (%)		
Urban	46 179 (75.8)	3207 (77.8)
Rural	14 747 (24.2)	916 (22.2)
Material deprivation quintile, *n* (%)		
Quintile 1 (most privileged)	10 326 (16.7)	736 (17.4)
Quintile 2	11 084 (17.9)	736 (17.4)
Quintile 3	11 601 (18.7)	798 (18.8)
Quintile 4	12 710 (20.5)	828 (19.5)
Quintile 5 (most deprived)	12 710 (20.5)	862 (20.3)
Missing	3459 (5.6)	282 (6.6)
Social deprivation quintile, *n* (%)		
Quintile 1 (most privileged)	10 842 (17.5)	692 (16.3)
Quintile 2	11 725 (18.9)	773 (18.2)
Quintile 3	12 506 (20.2)	832 (19.6)
Quintile 4	11 929 (19.3)	810 (19.1)
Quintile 5 (most deprived)	11 429 (18.5)	864 (20.4)
Missing	3459 (5.6)	271 (6.4)
Hypertension, *n* (%)	31 567 (51.0)	1707 (40.2)
Chronic kidney disease, *n* (%)	3826 (6.2)	264 (6.2)
Diabetes, *n* (%)	8333 (13.5)	614 (14.5)
Use of aspirin, *n* (%)	26 416 (42.7)	1337 (31.5)
Use of anticoagulants, *n* (%)	5477 (8.8)	403 (9.5)
Use of other lipid‐lowering drugs, *n* (%)	3274 (5.3)	255 (6.0)
Blood pressure treatment, *n* (%)	44 724 (72.3)	2460 (58.0)
Comorbidity score, mean (SD)	1.9 (2.9)	1.9 (3.2)
Number of medications in previous year, mean (SD)	9.0 (5.4)	8.1 (5.5)
Number of visits to a general practitioner in previous year, mean (SD)	1.1 (1.4)	1.0 (1.5)
Number of visits to a specialist in previous year, mean (SD)	2.7 (5.7)	2.8 (6.8)
Number of visits to an emergency in previous year, mean (SD)	0.3 (0.8)	0.3 (0.8)
≥ 1 visit for an emergency in previous year, *n* (%)	10 926 (17.7)	722 (17.0)
Number of days of hospitalization in previous year, mean (SD)	0.1 (0.3)	0.1 (0.3)
≥ 1 day of hospitalization in previous year, *n* (%)	3936 (6.4)	232 (5.5)

**FIGURE 2:**
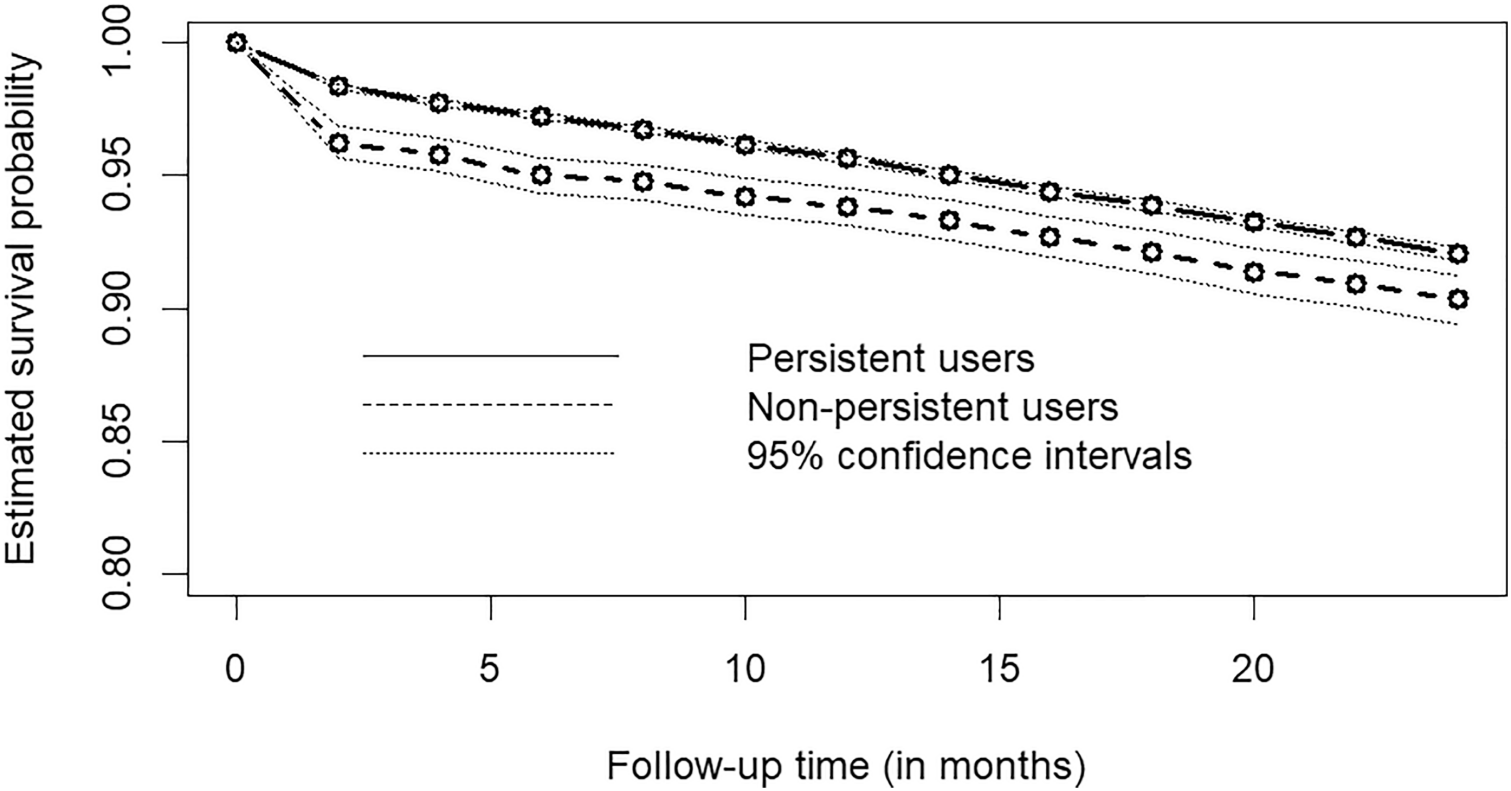
Counterfactual survival probabilities estimated by TMLE with Super Learner.

Table 3 reports the estimated hazard ratios using a traditional Cox model approach and TMLE with Super Learner. Both approaches lead to similar conclusions, wherein statin persistence for at least three months is associated with a reduction of the hazard of cardiovascular event or death, although the extent of the estimated reduction varies between approaches. Figure 2 represents the estimated survival curves for each exposure group. It shows that both groups have a relatively large drop in survival within the first two months, but the drop is much larger in the non‐persistent group than in the persistent group. The survival probability then decreases at a similar rate within both groups.

**TABLE 3:** Estimated hazard ratio of a first cardiovascular event or death according to statin persistence among new users aged 66 or more in Quebec, Canada.

Method	HR (95% CI)
Traditional Cox model	0.69 (0.63, 0.76)
TMLE with super learner	0.81 (0.73, 0.89)

*Note:* HR = Hazard ratio, CI = Confidence interval.

## 7 Discussion

Statistical methods for estimating causal effects have been developing at an extremely fast rate in the last few decades. It is thus challenging for analysts interested in this topic to remain up‐to‐date. This challenge is accentuated by the fact that new methods are generally first introduced in articles targeting a theoretical statistics audience. Such articles are rarely accessible to analysts who focus more on applications. In this article, we have presented a tutorial for using targeted maximum likelihood estimation to estimate the causal effect of a point exposure on a time‐to‐event outcome. More specifically, our tutorial explained how to use TMLE to estimate counterfactual survival curves and the parameters of a working marginal structural model. To the best of our knowledge, no tutorial on this topic was yet available. Our article adds to a growing literature of tutorials concerning the use of TMLE in specific settings [7, 12, 13, 14, 15].

Our tutorial aimed to provide an intuition of how TMLE operates to estimate a causal effect with a time‐to‐event outcome, while providing enough detail to allow readers with the required skills to implement the method on their own and to adapt the implementation to specifics of their application (e.g., with a different time ordering of variables than the one we considered). We have also provided boxes with example R code implementing TMLE in a simple illustration based on simulated data to facilitate replication. To further facilitate the implementation of TMLE, we have also provided an R function, available on GitHub (https://github.com/detal9/SurvTMLE). This GitHub repository includes additional documentation for the R function we have developed as well as supplementary examples based on simulated data. While our tutorial focused on the case of a binary exposure, the R function we provide also supports categorical exposures. Our article also included a guidelines section discussing when it is appropriate to use TMLE to estimate a causal effect as well as best practices for implementing TMLE. Finally, we have provided an illustration with real data. In this illustration, TMLE with machine learning produced a hazard ratio estimate suggesting less benefit from statin persistence than the traditional Cox model, which is more consistent with previous results of randomized controlled trials (OR for all‐cause mortality = 0.86 [95% CI: 0.79, 0.94], RR for cardiovascular disease = 0.75 [95% CI: 0.67, 0.80]) [66]. TMLE also allowed for the estimation of the counterfactual survival curve.

Some limitations of our tutorial also need to be taken into consideration. First, we have only considered the case of a single exposure measured at a single time‐point. TMLE can also be used with multiple exposures or with a time‐varying exposure. Petersen et al. have proposed TMLE algorithms adapted to such cases [40]. While we propose an R function implementing TMLE for a time‐to‐event outcome, we also recognize that various other implementations of TMLE are available, such as the R package ltmle. This package is very flexible, allowing to estimate the effect of fixed or dynamic time‐varying exposure regimes on a time‐varying outcome, with or without censoring. Because the ltmle package fits more generic purposes, some analysts may however find it easier to use our proposed R function than ltmle. Our proposed function also includes features specific to the time‐to‐event context that are not available in the ltmle package, such as modeling the counterfactual hazards with an MSM. We have also focused on a specific TMLE algorithm for time‐to‐event outcomes, but several other algorithms have been developed [40, 42, 43, 44, 67, 68, 69, 70]. Among others, TMLE algorithms that accommodate competing risks [44, 71], or that do not require discretizing the follow‐up time are available [42, 43, 44]. The R package survtmle available on https://github.com/benkeser/survtmle allows for the estimation of adjusted cumulative incidences with or without competing risks using TMLE [71]. Notably, the TMLE algorithm we have considered requires a discretization of the follow‐up time, which can be a challenging task and may lead to bias if discretization is too coarse [51]. A coarse discretization may however be required if sample size is small to avoid having sub‐periods without any event, which may lead to increased bias in a direction that is challenging to anticipate. Finally, the variance estimators we have used are based on the efficient influence function and are only valid if the exposure, censoring and outcome models are all estimated consistently at adequate convergence rates, either using not too adaptive approaches (i.e., Donsker condition) [37] or using cross‐fitting [54]. While this may seem like an important limitation of TMLE as compared to alternative approaches, TMLE allows the incorporation of machine learning methods to flexibly model the exposure, censoring and outcome. When machine learning approaches are used within TMLE, the modeling assumptions that need to be made are much more limited than when parametric models are employed. For instance, many machine learning algorithms can readily accommodate complex interactions and non‐linear relations. These variance estimators are also sensitive to near‐positivity violations. To some extent, issues related to near‐positivity violations can be controlled in an ad‐hoc fashion by truncating exposure and censoring probabilities using the gbound option in our R function. Variance estimators that are more robust to near‐positivity violations have recently been introduced [38]. Future work includes the expansion of the R function we have supplied. We aim to implement the novel variance estimators that are more robust to near‐positivity violations as well as cross‐fitting capabilities in the future [38, 54]. Extensions to time‐varying treatments and to permit competing risks will also be considered.

Some limitations of TMLE itself should also be noted. While using machine learning algorithms can help to better control for measured confounders, it does not help control for unmeasured (or poorly measured) confounders. The bias attributable to unmeasured confounders can often be expected to be much greater than the bias due to incorrect modeling of measured confounders. In addition, the TMLE algorithm is more complex than many alternatives, particularly propensity score approaches. This additional complexity can come at the cost of greater computational burden and poorer scalability to large databases, especially when machine learning algorithms are used.

In conclusion, we trust that this tutorial will be helpful to various kinds of readers, whether statisticians who are looking for a gentle theoretical introduction to TMLE or to analysts who aim to use TMLE to analyze their data. Given its theoretical benefits as compared to traditional approaches, we strongly encourage analysts to give TMLE a try.

## Conflicts of Interest

The authors declare no conflicts of interest.

## Supporting information

Data S1.

## Data Availability

The R code used for performing the simulation study is available on GitHub (https://github.com/detal9/SurvTMLE) and as supplementary Data Files.

## Appendix A Iterated Nested Expectation

In this appendix, we demonstrate how the counterfactual survival probability $S^{a}(t)$ is identified from the observed data using iterated nested expectations. These iterated nested expectations are at the core of Algorithm 1.

First, note that $S^{a}(t)=P(Y_{t}^{a,C_{t}=0}=0)=1-P(Y_{t}^{a,C_{t}=0}=1)=1-E[Y_{t}^{a,C_{t}=0}]$. Indeed, the counterfactual probability of remaining event‐free until time t ($S^{a}(t)$) can equivalently be represented as the counterfactual probability of having no event at time t or before time t in a hypothetical population where censoring is prevented ($P(Y_{t}^{a,C_{t}=0}=0)$). Adding the condition that censoring is prevented ($C_{t}=0$) is necessary because the event indicator $Y_{t}$ is missing if there is censoring.

We now demonstrate with details the identifiability for the case t=2, that is, we show that

$$\begin{array}{ll} & E\left(Y_{2}^{a,C_{2}=0}\right)=E\left\{E\left[E\left(Y_{2}|A=a,C_{2}=0,{\bar{L}}_{2}\right)|A=a,C_{1}=0,L_{1}\right]\right\} \end{array}$$

under the causal assumptions described in Section 3. First, using the rule of total expectations, we can write

$$\begin{array}{l} E\left(Y_{2}^{a,C_{2}=0}\right)=E\left[E\left(Y_{2}^{a,C_{2}=0}|L_{1}\right)\right] \end{array}$$

Next, appealing to the conditional exchangeability assumption ($Y_{t}^{a,C_{t}=0}\;⊥\;⊥\;A|L_{1}$ and $Y_{t}^{a,C_{t}=0}\;⊥\;⊥\;C_{k}|A,{\bar{L}}_{k},C_{k-1}=0$) we have

$$\begin{array}{ll} E\left[E\left(Y_{2}^{a,C_{2}=0}|L_{1}\right)\right] & =E\left[E\left(Y_{2}^{a,C_{2}=0}|A=a,L_{1}\right)\right]\\ & =E\left[E\left(Y_{2}^{a,C_{2}=0}|A=a,C_{1}=0,L_{1}\right)\right] \end{array}$$

Using once more the rule total expectations followed by the conditional exchangeability assumption, we get

$$\begin{array}{ll} & E\left[E\left(Y_{2}^{a,C_{2}=0}|A=a,C_{1}=0,L_{1}\right)\right]\\ & \;=E\left\{E\left[E\left(Y_{2}^{a,C_{2}=0}|A=a,C_{1}=0,{\bar{L}}_{2}\right)|A=a,C_{1}=0,L_{1}\right]\right\}\\ & \;=E\left\{E\left[E\left(Y_{2}^{a,C_{2}=0}|A=a,C_{2}=0,C_{1}=0,{\bar{L}}_{2}\right)\right.\right.\\ & \;\times \left.\left.|A=a,C_{1}=0,L_{1}\right]\right\} \end{array}$$

Because $C_{2}=0$ implies $C_{1}=0$, the last equality can be simplified to

$$E\left\{E\left[E\left(Y_{2}^{a,C_{2}=0}|A=a,C_{2}=0,{\bar{L}}_{2}\right)|A=a,C_{1}=0,L_{1}\right]\right\}$$

Finally, using the consistency assumption (i.e., A=a and $C_{t}=0\Rightarrow Y_{t}=Y_{t}^{a,C_{t}=0}$), we can write this last equation as

$$E\left\{E\left[E\left(Y_{2}|A=a,C_{2}=0,{\bar{L}}_{2}\right)|A=a,C_{1}=0,L_{1}\right]\right\}$$

which completes the demonstration.

The general proof proceeds similarly, appealing successively to the rule of total expectations and the conditional exchangeability assumption, and finally to the consistency assumption to show that

$$\begin{array}{ll} E\left(Y_{t}^{a,C_{t}=0}\right) & =E\left(E\left\{\cdots E\left[E\left(Y_{t}|A=a,C_{t}=0,{\bar{L}}_{t}\right)\right.\right.\right.\\ & \;\times \left.\left.\left.|A=a,C_{t-1}=0,{\bar{L}}_{t-1}\right]\cdots |A=a,C_{1}=0,L_{1}\right\}\right) \end{array}$$

Algorithm 1 implements this formula. Indeed, this algorithm first estimates the inner expectation $E\left(Y_{t}|A=a,C_{t}=0,{\bar{L}}_{t}\right)$ then produces doubly robust estimates of this quantity using TMLE. The updated predicted probabilities (${\hat{Q}}^{a}(t,\ell_{i,t})$) are then used as the outcome for the next iteration, which implements the second innermost expectation, that is,

$$E\left[\underset{Q^{a}(t,\ell_{i,t})}{\underbrace{E\left(Y_{t}|A=a,C_{t}=0,{\bar{L}}_{t}\right)}}|A=a,C_{t-1}=0,{\bar{L}}_{t-1}\right]$$

These steps are repeated until the outermost expectation is implemented by computing the sample mean of the updated predicted values ${\hat{Q}}^{a,*}(1,L_{1})$.

## Appendix B Derivation of the Influence Function of the Parameters of the Hazard Marginal Structural Model

The parameters of the hazard marginal structural models are defined as

$$\begin{array}{ll} & \gamma =\underset{\gamma }{\text{argmax}\;}\\ & \;E\left\{\underset{a,t}{\sum }\left[\log\left(\text{expit}{\left(X_{a,t}^{⊤}\gamma \right)}^{I(T^{a}=t)}{\left(1-\text{expit}(X_{a,t}^{⊤}\gamma )\right)}^{I(T^{a}>t)}\right)\right]\right\} \end{array}$$

The previous expectation can be written as

$$\begin{array}{ll} & \underset{a,t}{\sum }S^{a}(t-1)\left\{\frac{S^{a}(t-1)-S^{a}(t)}{S^{a}(t-1)}\log(\text{expit}(X_{a,t}^{⊤}\gamma ))\right.\\ & \;\left.+\left[1-\frac{S^{a}(t-1)-S^{a}(t)}{S^{a}(t-1)}\right]\left[\log(1-\text{expit}(X_{a,t}^{⊤}\gamma ))\right]\right\} \end{array}$$

The score equation is obtained by taking a derivative of the previous expression according to γ and is

$$\begin{array}{l} U(S,\gamma )=\underset{a,t}{\sum }S^{a}(t-1)X_{a,t}\left[\frac{S^{a}(t-1)-S^{a}(t)}{S^{a}(t-1)}-\text{expit}(X_{a,t}^{⊤}\gamma )\right] \end{array}$$

Using the functional delta method and the implicit function theorem, the efficient influence function of γ is

$$\begin{array}{l} IC_{\gamma }=-\underset{a,t}{\sum }{\left[\frac{\partial U(S,\gamma )}{\partial \gamma }\right]}^{-1}\frac{\partial U(S,\gamma )}{\partial S^{a}(t)}IC_{a}(t) \end{array}$$

where

$$\begin{array}{ll} \frac{\partial U(S,\gamma )}{\partial \gamma } & =\underset{a,t}{\sum }S^{a}(t-1)\frac{\exp(X_{a,t}^{⊤}\gamma )}{{\left[1+\exp(X_{a,t}^{⊤}\gamma )\right]}^{2}}X_{a,t}X_{a,t}^{⊤}\\ \frac{\partial U(S,\gamma )}{\partial S^{a}(t)} & =-X_{a,t}+X_{a,t+1}{\left[1+\exp(X_{a,t+1}^{⊤}\gamma )\right]}^{-1} \end{array}$$
